# Resolutions

**Published:** 1914-03-15

**Authors:** Herbert Sands Sutphen, Alphonso Irwin, William E. Truex, Vernon D. Rood, Cornelius Kiel, Charles P. Tuttle


### RESOLUTIONS.

On the death of Charles A. Meeker, D.D.S., Ex-Secretary of the N. J. State Board of Registration and Examination in Dentistry.

---

WHEREAS, In the providence of an All-Wise Creator our fellow-member, Dr. Charles A. Meeker died September 8th, 1913, and

WHEREAS, His indefatigable industry, his record as an examiner, his skill as an organizer and systematic devel oper of the New Jersey State Board work for a period of time covering twenty years, has placed him without a peer among eminent examiners;

NOW THEREFORE BE IT RESOLVED, That we, the members of the New Jersey State Board of Registration and Examination in Dentistry, deeply deplore his loss, and express our great appreciation of his long and intelligent services; and be it further

RESOLVED, That a page of our Minute Book be inscribed with these Resolutions and a copy of the same be sent to his bereaved widow and to the professional journals for publication.

*Signed* Herbert Sands Sutphen, D.D.S., President.
Alphonso Irwin, D.D.S., Secretary.
William E. Truex, D.D.S.
Vernon D. Rood, D.D.S.
Cornelius Kiel, D.D.S.
Charles P. Tuttle, D.D.S.

